# Association between Retinal Nerve Fiber Layer Thickness and Eye Fatigue

**DOI:** 10.1155/2019/3014567

**Published:** 2019-01-23

**Authors:** Masahiko Ayaki, Manami Kuze, Mineo Kondo, Kazuo Tsubota, Kazuno Negishi

**Affiliations:** ^1^Department of Ophthalmology, Keio University School of Medicine, Japan; ^2^Otake Clinic Moon View Eye Center, Japan; ^3^Department of Ophthalmology, Mie University School of Medicine, Japan; ^4^Division of Ophthalmology, Matsusaka General Hospital, Japan

## Abstract

Eye fatigue is a common health problem across all age groups. Herein, we explored the correlation between eye fatigue and thickness of the retinal nerve fiber layer (NFL). Included in the NFL are intrinsically photosensitive retinal ganglion cells (ipRGCs), which are associated with trigeminal pain. This retrospective cross-sectional study included outpatients with best-corrected visual acuity above 20/30 in both eyes and without dry eye, glaucoma, or retinal disease. A total of 1981 patients were initially enrolled and 377 patients were declared as eligible for the study analysis. We tested subjects for the presence of major ocular symptoms and measured thickness of ganglion cell complex (GCC) using optical coherence tomography. A total of 377 outpatients (46.4% men, mean age of 57.1 years) were enrolled for analysis, based on the interview-reported prevalence of six eye symptom, as follows: 31.5% for eye fatigue, 19.2% for blurring, 18.6% for dryness, 15.7% for photophobia, 13.5% for irritation, and 4.6% for pain. The macular GCC was significantly thicker in subjects with eye fatigue compared to the group not reporting eye fatigue (103.8 *μ*m versus 100.3 *μ*m,* P *= 0.014). Regression analysis identified eye fatigue (*P = *0.026, *β*=0.122, adjusted for age and sex) and dryness (*P *=0.024, *β*=0.130) as significantly correlated with the macular GCC thickness, while the full macular thickness showed no significant correlation. In conclusions, eye fatigue and dryness were positively associated with thickness of the macular GCC. Nonvisual symptoms might therefore play a role in the development of eye fatigue.

## 1. Introduction

Eye fatigue can be a serious problem for people of any age. Even in the absence of an ocular disorder, many people feel eye fatigue during intensive and near visual tasks or light exposure, and it is exacerbated in cases of dry eye disease (DED) [1, 2]. Eye fatigue in individuals with normal vision is mostly due to inappropriate spectacle correction, excessive visual load, and DED and might therefore be relieved by refractive correction and DE treatments. DED-associated eye fatigue related to vision impairment might additionally be caused by decreased image quality due to unstable tear film and Rayleigh scattering of visible blue light (395-490 nm wavelength), with neuropathic pain also recently implicated in the pathology underlying DED [3–5]. Light also exacerbates corneal pain and melanopsin-containing intrinsically photosensitive retinal ganglion cells (ipRGCs) can be a primary circuit for light aversion, with extensive investigations conducted to define their projections and functions. The ipRGCs projections include brain [6–12] and other retinal neurons [13–16] and the ciliary body [17]. The function of the ciliary body projections is unknown, with suggestions that they contribute to a small degree of pupil constriction mediated by melanopsin-containing ipRGCs located in the iris itself [18]. Corneal pain has not been directly associated with these melanopsin-expressing cells, but it potentially could be mediated by melanopsin-expressing trigeminal neurons and further studies are needed to support the current evidence ([19–24].

This signaling system provides another possible explanation for the protective effects of blue-light shield eyewear against DED-associated and general eye fatigue. Eye closure and darkness are the most effective ways to reduce or avoid eye fatigue through reducing dryness of the ocular surface and relieving the visual load, while shading of blue-light and reducing light scattering are sufficiently effective in reducing eye fatigue [25–27]. The ipRGCs have been proposed to cause deep ocular pain and photophobia, both of which are major symptoms of eye fatigue. Reduced activation of ipRGCs by blue-light shield shading could also be as effective at relieving eye fatigue as eye closure and darkness, via the photophobic association of ipRGCs [28–30].

Thickness of the retinal nerve fiber layer (NFL), which includes ipRGCs, is now easily measured in eye clinics using optical coherence tomography (OCT), which is noninvasive, rapid, and highly reproducible. NFL thickness has also been used by ophthalmologists to diagnose glaucoma and positively associated with ipRGC activity [31]. Finally, OCT has been tested in psychiatric and cerebellar disorders to explore possible associations between retinal thickness and brain function [32].

We therefore hypothesized that ipRGCs might be involved in the development of common ocular symptoms since these cells are associated with both visual and nonvisual responses; however, human data are limited to pupillary responses in retinal degeneration, cataract, and glaucoma, as well as electroretinography findings for glaucoma [33]. This study explored the association between retinal thickness and common eye symptoms in apparently normal eyes. We excluded subjects with short tear break-up time (TBUT) and diffuse keratoepitheliopathy because subjects with suspected DED could present various symptoms that might confound the interpretation of results.

## 2. Methods

### 2.1. Study Institutions and Institutional Review Board Approval

Outpatients were consecutively recruited to the study from January 2014 to March 2017 from six general eye clinics in Japan. The Institutional Review Boards and Ethics Committees of Shinseikai Toyama Hospital (Permit Number: 150503) and Komoro Kosei General Hospital (Permit Number: 2705) approved this study, and the study was performed in accordance with the principles of the Declaration of Helsinki. Informed consent was obtained from all participants.

### 2.2. Recruitment of Patients with Eye Fatigue

**Figure 1:**
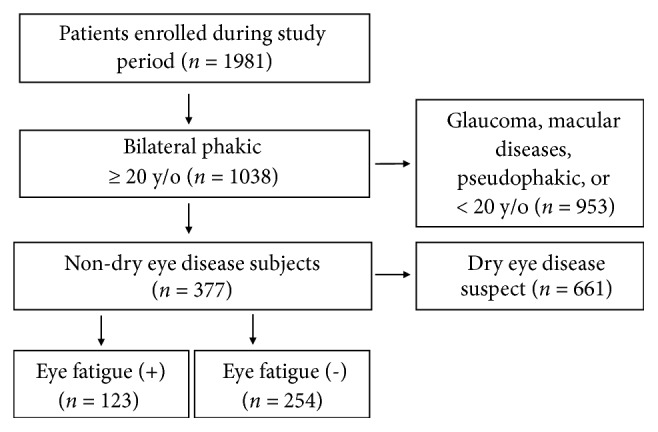
Flow diagram of the patient enrolment and inclusion process. Details of the inclusion and exclusion criteria are provided in the text. OCT, optical coherence tomography.

A total of 1981 patients were initially enrolled during the study period. Following application of the inclusion and exclusion criteria, 254 patients without eye fatigue and 124 patients with eye fatigue were declared as eligible for the study analysis (Figure 1). Inclusion criteria were consecutive outpatients aged over 19 years with best-corrected visual acuity better than 20/30 in both eyes. Exclusion criteria were any ocular surgery within one month, short TBUT (≤ 5 s), diffuse keratoepitheliopathy disturbing the optical axis, glaucoma treated with medication, any macular disease including age-related macular degeneration, diabetic retinopathy, and epiretinal membrane, and any acute eye disease within one week. Consequently, the final study cohort predominantly comprised individuals visiting their clinic for an annual eye examination or for outer adnexal eye disease.

None of the patients had undergone any nonmedical interventions, such as punctal plug insertion or punctal occlusion, or any surgical interventions. In six patients, Sancoba^R^ eye drops (cyanocobalamin; Santen Pharmaceutical Co. Ltd, Osaka, Japan) were prescribed for the treatment of eye fatigue.

### 2.3. Patient Interviews for Common Eye Symptoms

Participants were first interviewed regarding major ocular symptoms related to eye fatigue to determine the presence or absence (yes/no) of six common ocular symptoms, namely eye fatigue, blurring, photophobia, pain, dryness, and irritation. These symptoms were selected as the six most prevalent of outpatients visiting the eye clinic of Keio University Hospital in 2012.

### 2.4. Ophthalmological Examinations

Board-certified ophthalmologists with specialist expertise in retinal, glaucoma, and corneal disorders submitted all subjects to a routine examination comprising visual acuity and intraocular pressure testing, biomicroscopy with vital corneal staining, and ophthalmoscopy. Examinations were also conducted to exclude DED according to the Asia Dry Eye Society [34], which defines DED as the presence of a short TBUT (≤ 5 s) and DED-related symptoms. We also tested subjects by the Schirmer test with anesthesia (≤ 5 mm), maximum blinking interval (MBI) (≤ 9 s), and vital corneal staining. The MBI was expressed as the number of seconds the eyes could stay open without blinking.

A blinded examiner measured binocular near add power at a distance of 30 cm using a Bankoku near-acuity chart (Handaya Inc., Tokyo, Japan) or an automatic optometry system (AOS-700^R^; Nidek, Gamagori, Japan). After determining the patient's distance refractive correction, the minimal additional power required to achieve near acuity better than 20/25 was measured in 0.25-D increments and recorded as near add power.

#### 2.4.1. OCT Measurement

Spectral domain OCT data were obtained using the RS 3000^R^ (Nidek Co.ltd., Aichi, Japan), and all OCT imaging was performed using the raster-scan protocol. Data obtained during apparent eye movements, influenced by involuntary blinking or saccade, or with a Signal Strength index < 7 were excluded, as recommended by the manufacturer. The macular ganglion cell complex (GCC; retinal nerve fiber layer (RNFL) + ganglion cell layer (GCL) + inner plexiform layer (IPL)) diameter of 9 mm and the full retinal thickness in the central macular area diameter of 1 mm were analyzed as follows. The fovea was automatically identified as the pixel with the least retinal thickness close to the fixation point, and a square imaging area (9 × 9 mm) was centered on the fovea. Using software supplied from the manufacturer, the thicknesses of (i) NFL, (ii) GCL +IPL, (iii) internal limiting membrane (INL) + outer plexiform layer (OPL), (iv) ONL + inner segment layer (IS), and (v) outer segment layer (OS) + retinal pigment epithelium (RPE) were exported as a pixel image (512 × 128 pixels), and the mean thickness values of the whole analysis area (9.0 × 9.0 mm, corrected for axial length) and excluding the optic disc and peripapillary atrophy were calculated.

### 2.5. Statistical Analysis

Where appropriate, data are given as the mean ± SD. We analyzed the data from the right eye for TBUT, Schirmer test, refraction, and the full retinal thickness of whole macula. To identify which ophthalmic parameters were correlated with the six symptoms, regression analysis was performed with potential symptoms including eye fatigue used as dependent variables, while demographic (age and sex) and ophthalmic parameters (OCT, refraction, DE-related corneal parameters) were used as independent variables. The regression line was computed for age and left superior macular GCC thickness of subjects with and without eye fatigue by the least-square method. Pearson's correlation coefficient was used as a measure of association between age and left superior macular GCC. The difference in two regression line slopes was analyzed by t-test. All analyses were performed using StatFlex^R^ (Atech, Osaka, Japan) with* P *< 0.05 considered significant.

## 3. Results

### 3.1. Results of Ocular Symptomatology and Retinal Thickness

A total of 377 outpatients (46.4% men, mean age of 57.1 ± 16.8 years, 20-93 years) were enrolled for analysis. Prevalence of the six symptoms reported by interview was 31.5% for eye fatigue, 19.2% for blurring, 15.7% for photophobia, 18.6% for dryness, 13.5% for irritation, and 4.6% for pain. Before exclusion of suspected DED cases (n = 661) from the cohort (Figure 1), 356 (34.3%) of 1038 subjects reported eye fatigue, and 222 (62.4%) had short TBUT or keratoepitheliopathy.

**Figure 2:**
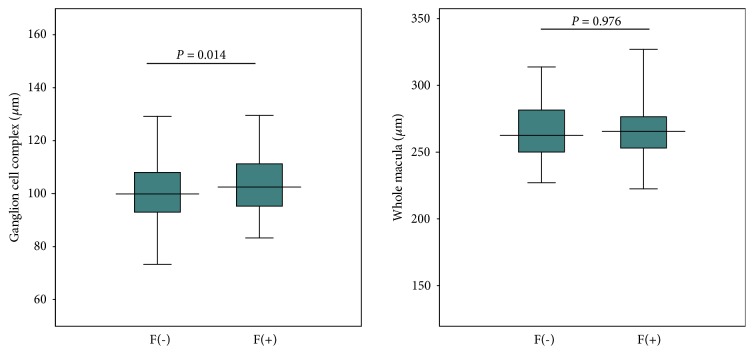
Box plots showing the distribution of retinal thickness in subjects with and without eye fatigue. There was a significant difference in mean macular ganglion cell complex thickness between subjects with and without eye fatigue (*P *= 0.014, unpaired t-test) (left panel), while the full macular thickness was not different between groups (*P *= 0.976) (right panel). The horizontal line in each diagram indicates the median value. The height, positive error bar, and negative error bar of each box indicate the 25th–75th percentiles, maximum values, and minimum values, respectively. F(-) = without eye fatigue; F(+) = with eye fatigue.

We next compared each parameter between subjects with and without eye fatigue (Table 1). The other five reported symptoms were also more prevalent in the subjects reporting eye fatigue compared to those without it, and MBI was shorter in the eye fatigue group than in the noneye fatigue group. In contrast, the Schirmer test result, refractive and near add power were not different between groups. Finally, mean thickness of the macular GCC was significantly larger in subjects with eye fatigue than in those without in all hemispheres except for the superior right (Figure 2), whereas the full macular thickness was not different between groups. The difference in GCC thickness was most prominent in left superior hemisphere (*P*=0.008). The results of comparison of ocular surface parameters and retinal thickness between subjects with and without the other five symptoms are shown in Table 2. The mean thickness of the macular GCC was significantly larger in subjects with dryness than in those without (*P*=0.007), whereas there was no difference for the other symptoms. The full macular thickness was not different between groups.

**Figure 3:**
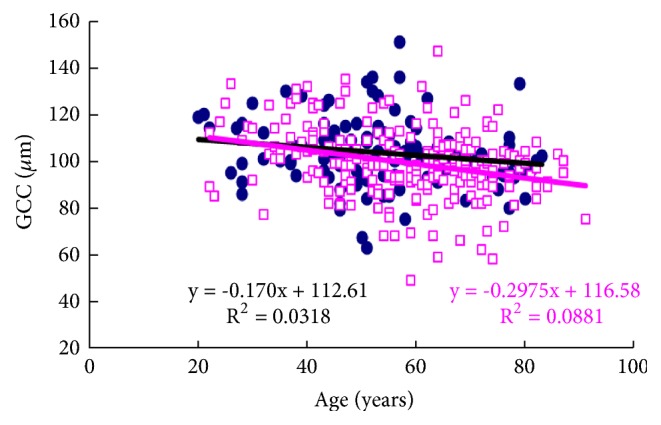
Scatter plots and regression lines of age-related thinning of superior left macular ganglion cell complex (GCC). The annual decrease in GCC thickness was not significantly larger in subjects with eye fatigue (0.30 *μ*m) than in those without (0.17 *μ*m;* P* = 0.222). Black closed circles and black regression lines indicate subjects with eye fatigue, and red open squares and red regression lines indicate subjects without eye fatigue.

The regression analysis for ocular symptoms and retinal thickness identified eye fatigue and dryness as significantly correlated with the thickness of GCC in six symptoms, while full retinal thickness of the whole macula was not correlated with any symptom by linear or multiple regression analysis (Table 3). Scatter plots and regression lines of age-related thinning of superior left macular GCC indicated that annual decrease in GCC thickness was not significantly larger in subjects with eye fatigue (0.30 *μ*m) than in those without (0.17 *μ*m) (*P* = 0.222) (Figure 3).

## 4. Discussion

The present study demonstrated a significant correlation between eye fatigue and thickness of the macular GCC, but not the full macula thickness, suggesting that ipRGCs contained within the GCC could have a role in the development of eye fatigue. In such a scenario, subjects with a thick macular GCC might feel eye fatigue with exposure to blue-light emitting lamps and displays. This analysis thus proposes a unique insight into the pathophysiology of eye fatigue whereby subclinically decreased photoreception and visual function might be involved in developing eye fatigue, potentially accounting for the universal effectiveness of eye closure in relieving eye fatigue. Interestingly, younger subjects with less presbyopia reported more eye fatigue and this might be related to their higher intraocular light transmittance [35] and thicker GCC. Thus, eye fatigue could act as a defense mechanism protecting the eye from excessive exposure to light. Indeed, it is well known that many patients complain of eye fatigue while opening their eyes even without watching anything. This study did not show an association between photophobia and GCC thickness; however, photophobia is a multifactorial manifestation in human patients [28–30], and GCC alone might not be a contributing factor.

Age-thickness plotting showed a similar annual decrease across the two groups (Figure 3). Kita et al. [36] reported a mean thickness of macular GCC of 98.08 ± 7.88 *μ*m in the superior hemisphere and 98.57 ± 7.64 *μ*m in the inferior hemisphere for a Japanese population, while Ooto et al. [37] described a mean decrease in GCC of 0.17 *μ*m/year in Japanese subjects, comparable with our results. The eye fatigue group was statistically 4 years younger, implicating an estimate of 0.68 *μ*m thickness difference. Thus we speculate that the difference in GCC thickness between the groups was significant, and thus hypothesize that lower amounts of degeneration, edema, and scarring of retinal neurons are implicated in increased GCC volume for patients with eye fatigue.

Migraine and eye fatigue share the common symptom of allodynia (photophobia) and thus might be evoked by the trigeminal circuit driven by ipRGC activity. Allodynia in migraine is also evoked by many other triggers including heat and touch. Lack of insular thinning with age was described in female migraineurs compared with nonmigraineurs [38], while insula was associated with both pain and emotion and insular hyperexcitability was possibly apparent in migraineurs. We therefore speculate that subjects with a thicker than average GCC might experience photophobia as eye fatigue in a similar fashion.

Dryness was also significantly correlated with the macular GCC thickness, despite no significant correlation with a short BUT and the higher Schirmer test results in subjects with eye fatigue. Dryness in such cases is seemingly not due to corneal pathologies and we have no explanation thus far for the correlation with GCC thickness, except that subjects with corneal hyperesthesia can report dryness [5, 39] even with normal Schirmer test values and TBUT. The majority (62.4%) of eye fatigue subjects in our cohort had DED and their symptoms might therefore have reflected numerous factors including photosensitivity. Additionally, our previous survey demonstrated eye fatigue and dryness as the two most frequent symptoms in DED patients compared with non-DED controls [39]. There are multiple stages in corneal dysesthesia and neuropathic pain depending on corneal inflammation and neurodegeneration [5, 39]. Likewise, it is difficult to determine origins of dryness since corneal sensitization, pain, and ipRGC-mediated photosensitivity can overlap, thus etiology-based structured questionnaires and examinations would enable us to better characterize ocular symptoms with respect to eye fatigue and dryness [40].

**Figure 4:**
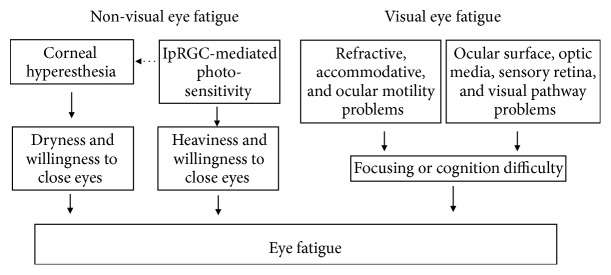
Schematic presentation of our proposed classification process for eye fatigue. We hypothesize that eye fatigue originates from both visual and nonvisual etiologies, with corneal hyperesthesia and photosensitivity playing major roles in nonvisual eye fatigue. Recent findings of neuropathic pain and ipRGCs underlie this classification model, whereby corneal pain might be mediated by ipRGCs, although the detailed neural mechanisms of dryness and photosensitivity remain elusive. ipRGCs, intrinsically photosensitive retinal ganglion cells.

Subjects with eye fatigue report a wide variety of symptoms including tiredness, focusing difficulty, blurring, brightness, dryness, foreign body sensation, headache, neck and shoulder pain, mental stress, glare, heaviness, and itching [1], as also shown in the present study. In addition, symptoms and pathophysiology are sometimes discordant in such individuals. Herein we propose a newly organized concept of eye fatigue according to the present results and recent advances in characterizing ipRGCs and the neural aspects of DED (Figure 4). Conventional understanding for eye fatigue has focused on the various modes of discomfort in and around the seeing eye. In contrast, we now propose that eye fatigue originates from visual and nonvisual pathophysiology. Corneal pain can be mediated by ipRGCs [19–24], although the detailed neural mechanisms of dryness and photosensitivity remain elusive. We thus recommend that eye-care practitioners also consider nonvisual eye fatigue in their patients, since it is historically overlooked.

This study has some limitations. The present patient population may include subclinical DED even after exclusion of short TBUT and keratoepitheliopathy cases, as it is known that eye fatigue and corneal dryness present heterogeneously and that treatments have varying efficacy, suggesting a complexity beyond simple correlations. Visual acuity corrected with participants' spectacles should have been examined since unsuitable correction is a major cause of eye fatigue. Eye pain should also be further evaluated with a validated questionnaire (e.g., Short-form McGill Pain Questionnaire) and esthesiometers. Of note, the anatomy, physiology, and function of human ipRGCs remain unclear and further studies are needed to determine how ipRGC activity levels might contribute to visual and nonvisual symptoms in humans. The difference in GCC thickness between groups should be further confirmed with quantitative pupillary light reflex measurements by direct measurement of ipRGC function.

## 5. Conclusions

Eye fatigue was positively associated with thickness of the macular GCC. We thus hypothesize that trigeminal activation might occur in conditions with photophobia/photoallodynia as a presenting symptom of eye fatigue, involving systems that alter melanopsin-based signaling without specification of the originating cell types including retinal, iris, and trigeminal. Nonvisual symptoms might therefore play a role in the development of eye fatigue.

## Figures and Tables

**Table 1:** Comparison of parameters between groups with and without eye fatigue.

Parameters	Eye fatigue (-)	Eye fatigue (+)	*P *value*∗*
No. of subjects	254	123	
Age (years)	58.4 ± 18.1	54.4 ± 15.8	0.013*∗*
% of men	55.9	48.8	0.196

Symptomatology			
Blurring (%)	15.0	28.5	0.004*∗*
Photophobia (%)	11.1	24.4	0.003*∗*
Pain (%)	2.4	8.9	0.018*∗*
Irritation (%)	8.7	23.6	0.001*∗*
Dryness (%)	11.4	33.3	< 0.001*∗*

Ocular surface parameters			
Schirmer test (% ≤ 5 mm)	21.7	13.3	0.511
Maximum blinking interval (% ≤ 9 s)	4.3	18.9	< 0.001*∗*

Refractive and accommodative parameters			
Spherical error (diopter)	–2.00 ± 3.14	–2.48 ± 3.36	0.209
Cylindrical error (diopter)	0.78 ± 0.71	0.84 ± 0.68	0.279
Spherical equivalent (diopter)	–2.39 ± 3.13	–2.84 ± 3.41	0.254
Anisometropia (diopter)	0.64 ± 0.77	0.65 ± 0.72	0.855
Near add power (diopter)	1.42 ± 0.18	1.49 ± 0.16	0.620

Retinal thickness			
Macular ganglion cell complex (*μ*m), mean	100.3 ± 13.4	103.8 ± 14.1	0.014*∗*
Superior right (*μ*m)	99.2 ± 15.4	103.0 ± 15.1	0.128
Superior left (*μ*m)	99.2 ± 16.2	103.4 ± 16.7	0.008*∗*
Inferior right (*μ*m)	101.4 ± 18.2	105.2 ± 18.6	0.033*∗*
Inferior left (*μ*m)	101.2 ± 18.1	105.3 ± 18.3	0.021*∗*
Full retinal thickness of whole macula (*μ*m)	266.1 ± 24.3	265.9 ± 26.1	0.976

*∗P *< 0.05, Chi squared test and t-test as appropriate.

**Table:** (a) Visual symptom

Parameters	Photophobia (-)	Photophobia (+)	*P *value*∗*	Blurring (-)	Blurring (+)	*P *value*∗*
No. of subjects (%)	318	68		303	73	
Age (years)	57.3 ± 14.7	56.0 ± 14.4	0.509	56.5 ± 15.2	59.8 ± 12.3	0.053
% of men	54.4	47.5	0.314	51.2	63.0	0.066

Schirmer test (% ≤ 5 mm)	20.0	16.7	0.912	18.8	16.7	0.912
MBI (% ≤ 9 s)	7.5	13.6	0.128	9.6	6.8	0.418

Macular GCC (*μ*m), mean	101.6 ± 13.4	100.6 ± 14.1	0.622	101.7 ± 12.8	99.9 ± 14.8	0.323
FRTWM (*μ*m)	266.0 ± 25.6	266.2 ± 25.5	0.980	265.7 ± 26.4	267.0 ± 22.0	0.842

**Table:** (b) Non-visual symptom

Parameters	Dryness (-)	Dryness (+)	*P *value*∗*	Pain (-)	Pain (+)	*P *value*∗*	Irritation (-)	Irritation (+)	*P *value*∗*
No. of subjects (%)	307	70		359	17		325	51	
Age (years)	57.9 ± 14.4	53.8 ± 15.1	0.045*∗*	57.5 ± 14.6	48.2 ± 14.3	0.018*∗*	56.9 ± 15.00	58.2 ± 12.58	0.334
% of men	54.7	48.6	0.358	52.9	64.7	0.349	54.7	48.6	0.205

Schirmer test (% ≤ 5 mm)	20.7	11.1	0.488	20.0	0	0.082	25.9	0	0.006*∗*
MBI (% ≤ 9 s)	8.5	11.6	0.462	8.7	17.6	0.366	7.8	14.5	0.242

Macular GCC (*μ*m), mean	100.6 ± 13.4	105.0 ± 11.5	0.007*∗*	101.3 ± 13.3	103.3 ± 10.5	0.461	101.4 ± 13.5	101.5 ± 11.3	0.966
FRTWM (*μ*m)	266.1 ± 20.3	265.9 ± 37.5	0.982	265.3 ± 25.0	272.6 ± 30.3	0.526	265.4 ± 26.2	270.4 ± 19.6	0.512

MBI, maximum blinking interval; GCC, thickness of macular ganglion cell complex; FRTWM, full retinal thickness of whole macula.

**Table 3:** Regression analysis of retinal thickness and ocular symptoms.

Linear regression						
	Symptoms					
Measured retinal thickness	Fatigue	Blurring	Photophobia	Dryness	Irritation	Pain
Macular ganglion cell complex	0.127	-0.056	-0.028	0.129	0.002	0.031
	(0.014*∗*)	(0.279)	(0.590)	(0.012*∗*)	(0.970)	(0.546)
Full thickness of whole macula	-0.003	0.020	0.003	-0.003	0.061	0.087
	(0.977)	(0.856)	(0.980)	(0.976)	(0.591)	(0.441)

Multiple regression^A^						
Measured retinal thickness	Fatigue	Blurring	Photophobia	Dryness	Irritation	Pain
Macular ganglion cell complex	0.122	-0.068	-0.043	0.130	-0.058	-0.003
	(0.026*∗*)	(0.199)	(0.413)	(0.024*∗*)	(0.311)	(0.956)
Full thickness of whole macula	0.032	0.003	0.018	-0.061	0.050	0.128
	(0.801)	(0.982)	(0.879)	(0.635)	(0.692)	(0.293)

Data show *β* values, with *P* values in parentheses. *∗P* < 0.05, adjusted for age and sex.

## Data Availability

The data used to support the findings of this study are available from the corresponding author upon request.
